# Supplementary material for: Association between serum uric acid and left ventricular hypertrophy/left ventricular diastolic dysfunction in patients with chronic kidney disease
Source: PLoS One. 2021 May 6;16(5):e0251333. doi: 10.1371/journal.pone.0251333 (PMC8101764; doi:10.1371/journal.pone.0251333)
Supplement: Attachment — Submitted filename: Response to Reviewers.docx [file pone.0251333.s001.docx]

**PONE-D-21-04044
Association between serum uric acid and left ventricular hypertrophy/left ventricular diastolic dysfunction in patients with chronic kidney disease**

**Editor’s comment**

Reviewer #1 raised a very important point. Thus, the definition of left-ventricular diastolic dysfunction is obviously crucial for this paper and should follow the current recommendations. Both reviewers indicated that previous research addressed the aim of this study already. This does not mean that it is not interesting but the authors need to put their work better into perspective and need to better address previous studies in relation to their own work. This should include proper discussion about the new aspects of their own work.

Answer)

First, we included the left atrium volume index in data analysis as reviewer #1’s comment

Second, as editor and reviewers comment, there has been a lot of research that demonstrated the adverse effect of SUA on LVH/LVDD. However, there have been only a few studies on the association between SUA and LVH/LVDD in the CKD population. Regarding the LVH in patients with CKD, Zeng et al. reported that elevated SUA levels were positively associated with an increased risk of LVH in CKD patients with type 2 diabetes [ J Diabetes Res. 2017; 2017: 5016093]. Yoshitomi et al. reported that SUA levels were associated with LVMI and LVH in female patients with CKD, whereas no such association was found in male patients with CKD [Hypertens Res. 2014; 37: 246-52]. In contrast to the two above-mentioned studies, the multivariable analysis in our study showed an association between SUA levels and LVH in patients with CKD, independent of diabetes or sex, suggesting that the SUA level is an independent predictor of LVH in the CKD population. Concerning LVDD in patients with CKD, Gromadzińsk et al. showed that hyperuricemia was an independent predictor of LVDD in 50 patients with CKD [Adv Clin Exp Med. 2015; 24: 47-54.]. However, their study was limited by the small sample size. Our study showed an independent association between SUA levels and LVDD in 1025 patients with CKD, a relatively large sample size meeting the statistical significance. Taken together, although our study is not the first study to investigate the association between SUA and LVH/LVDD in the CKD population, our study has the strengths over the previous studies in that it included a large number of patients with CKD (n = 1025) and assessed a variety of variables that could affect the LVH/LVDD in patients with CKD, such as intact PTH, phosphate, left atrial volume, etc. Thus, our study provides more solid evidence for the association between SUA and LVH/LVDD in the CKD population and raises awareness of the importance of SUA during the development of LVH/LVDD in the CKD population.

🡪This description was added in the discussion section of the revised manuscript

**Reviewer #1**

**MAJOR CONCERN**

Despite the authors have mentioned the analysis of the left atrial volume to classify the left ventricular diastolic function (Line 108), I was not able to find these results in the text. This is a cardinal data, recommended by the American Society of Echocardiography Guidelines for the Evaluation of Left Ventricular diastolic Function (Nagueh SF et al. J Am Soc Echocardiogr 2016;29:277-314). The authors need to show these results and to include them in the uni and multivariate analysis. There are studies showing that high levels of serum uric acid are associated with higher left atrial volumes (Sung, KT et al. Association of serum uric acid level and gout with cardiac structure, function and sex differences from large scale asymptomatic Asians. PLOSOne.2020; 15(7):e0236173; Pan KL et al. The effects of gout on left atrial volume remodelling: a prospective echocardiographic study. Rheumatology.2014 May;53(5):867-74). The left atrial volume is a predictor of cardiovascular events (Mancusi C et al. Left atrial dilatation: A target organ damage in young to middle-age hypertensive patients. The Campania Salute Network. Int J Cardiol. 2018;265:299-233) and it would be important to investigate and show the correlation between this parameter and serum uric acid levels in this group of patients.

Answer) As you suggested, we included left atrial volume index in tables and figure as follows

**Table 1. Baseline characteristics of the study population according to CKD stage (n = 1025).**

|  | **CKD stage 3**  **(n = 511)** | **CKD stage 4**  **(n = 356)** | **CKD stage 5**  **(n = 158)** | **P**^c^ |
| --- | --- | --- | --- | --- |
| Age (years) | 58.6 ± 9.9 | 61.2 ± 10.2 | 63.3 ± 12.0 | <0.001 |
| Sex, male [n (%)] | 262 (51.3%) | 189 (53.1%) | 88 (55.7%) | 0.841 |
| Diabetes [n (%)] | 259 (50.7%) | 178 (50.0%) | 85 (53.8%) | 0.721 |
| Cardiovascular disease [n (%)] |  |  |  |  |
| Coronary heart disease^a^ | 88 (17.2%) | 83 (23.3%) | 43 (27.2%) | 0.010 |
| Cerebrovascular disease^b^ | 40 (7.8%) | 41 (11.5%) | 31 (19.6%) | <0.001 |
| Peripheral vascular disease | 28 (5.5%) | 30 (8.4%) | 22 (13.9%) | 0.002 |
| Medication [n (%)] |  |  |  |  |
| ACEI or ARB | 373 (73.0%) | 274 (77.0%) | 131 (82.9%) | 0.033 |
| Calcium channel blocker | 298 (58.3%) | 236 (66.3%) | 110 (69.6%) | 0.009 |
| Beta blocker | 157 (30.7%) | 149 (41.9%) | 95 (60.1%) | <0.001 |
| Diuretics (thiazide) | 194 (38.0%) | 74 (20.8%) | 19 (12.0%) | <0.001 |
| Diuretics (loop) | 186 (36.4%) | 166 (46.6%) | 102 (64.6%) | <0.001 |
| Urate-lowering therapy | 75 (14.7%) | 76 (21.3%) | 47 (29.7%) | <0.001 |
| Gout | 67 (13.1%) | 60 (16.9%) | 35 (22.2%) | 0.020 |
| Body mass index (kg/m^2^) | 23.5 ± 2.5 | 23.6 ± 2.5 | 23.4 ± 2.0 | 0.716 |
| Systolic blood pressure (mmHg) | 129.6 ± 18.4 | 135.3 ± 18.4 | 142.3 ± 16.2 | <0.001 |
| Diastolic blood pressure (mmHg) | 79.7 ± 14.4 | 80.3 ± 14.5 | 81.3 ± 15.2 | 0.438 |
| eGFR (mL/min/1.73 m^2^) | 42.8 ± 8.5 | 22.2 ± 4.2 | 9.9 ± 3.4 | <0.001 |
| Urinary albumin (mg/g Cr) | 812.1 ± 818.2 | 1355.3 ± 1163.7 | 2241.0 ± 1421.9 | <0.001 |
| Albumin (g/dL) | 4.2 ± 0.4 | 4.1 ± 0.4 | 3.9 ± 0.5 | <0.001 |
| Uric acid (mg/dL) | 6.7 ± 2.7 | 7.6 ± 3.1 | 8.7 ± 2.9 | 0.004 |
| Calcium (mg/dL) | 9.1 ± 0.4 | 9.0 ± 0.4 | 8.9 ± 0.4 | <0.001 |
| Phosphate (mg/dL) | 3.4 ± 0.5 | 4.0 ± 0.8 | 4.7 ± 1.0 | <0.001 |
| Total cholesterol (mg/dL) | 208.8 ± 39.9 | 209.4 ± 41.4 | 207.6 ± 40.4 | 0.892 |
| Hemoglobin (g/dL) | 12.9 ± 1.7 | 10.9 ± 1.6 | 9.7 ± 1.5 | <0.001 |
| CRP (mg/dL) | 0.6 ± 0.5 | 0.7 ± 0.5 | 1.0 ± 0.9 | <0.001 |
| Intact PTH (pg/mL) | 56.5 ± 29.6 | 129.0 ± 74.0 | 196.2 ± 90.9 | <0.001 |
| LVMI (g/m^2^) | 95.0 ± 23.0 | 105.0 ± 24.3 | 113.8 ± 23.8 | <0.001 |
| E (cm/s) | 62.0 ± 8.4 | 61.2 ± 8.3 | 61.0 ± 10.2 | 0.230 |
| e' (cm/s) | 7.9 ± 1.4 | 7.6 ± 1.1 | 7.3 ± 1.3 | <0.001 |
| E/e' | 8.1 ± 1.7 | 8.2 ± 1.3 | 8.6 ± 2.4 | <0.001 |
| Left atrial volume index (mL/m^2^) | 34 ± 6.9 | 35.3 ± 6.6 | 36.1 ± 7.6 | 0.014 |
| LVEF (%) | 62.3 ± 6.7 | 61.3 ± 7.4 | 60.0 ± 7.6 | 0.001 |
| LVH | 153 (29.9%) | 167 (46.9%) | 105 (66.5%) | <0.001 |
| LVDD | 316 (61.8%) | 232 (65.2%) | 109 (69.0%) | 0.229 |

Data are presented as mean ± standard deviation or (n, %). ^a^Coronary heart disease is defined as a history of coronary artery bypass surgery or percutaneous transluminal coronary angioplasty. ^b^Cerebrovascular disease is defined as a history of stroke or transient ischemic attack. ^c^P indicates statistical significance between the 3 groups. ACEI, angiotensin-converting enzyme inhibitor; ARB, angiotensin receptor blocker; CKD, chronic kidney disease; CRP, C-reactive protein; E, early mitral inflow velocity; e', peak early mitral annular velocity; eGFR, estimated glomerular filtration rate; PTH, parathyroid hormone; LVDD, left ventricular diastolic dysfunction; LVEF, left ventricular ejection fraction; LVH, left ventricular hypertrophy; LVMI, left ventricular mass index.

🡪Patients with a higher CKD stage showed higher levels of left atrial volume index.

**Table 2. Baseline characteristics of the study population according to tertiles of serum uric acid values (n = 1025).**

|  | **Tertile 1**  **(<5.5 mg/dL)** | **Tertile 2**  **(5.6-8.4 mg/dL)** | **Tertile 3**  **(>8.4 mg/dL)** | **P**^c^ |
| --- | --- | --- | --- | --- |
| Age (years) | 59.0 ± 10.0 | 59.8 ± 10.4 | 61.9 ± 10.8 | 0.001 |
| Sex, male [n (%)] | 170 (50.1%) | 196 (57.6%) | 173 (50.0%) | 0.073 |
| Diabetes [n (%)] | 182 (53.7%) | 171 (50.3%) | 169 (48.8%) | 0.430 |
| Cardiovascular disease [n (%)] |  |  |  |  |
| Coronary heart disease^a^ | 61 (18.0%) | 75 (22.1%) | 78 (22.5%) | 0.276 |
| Cerebrovascular disease^b^ | 31 (9.1%) | 37 (10.9%) | 44 (12.7%) | 0.325 |
| Peripheral vascular disease | 22 (6.5%) | 24 (7.1%) | 34 (9.8%) | 0.218 |
| Medication [n (%)] |  |  |  |  |
| ACEI or ARB | 250 (73.7%) | 265 (77.9%) | 263 (76.0%) | 0.441 |
| Calcium channel blocker | 208 (61.4%) | 213 (62.6%) | 223 (64.5%) | 0.701 |
| Beta blocker | 125 (36.9%) | 131 (38.5%) | 145 (41.9%) | 0.387 |
| Diuretics (thiazide) | 108 (31.9%) | 98 (28.8%) | 81 (23.4%) | 0.137 |
| Diuretics (loop) | 143 (42.2%) | 145 (42.6%) | 166 (48.0%) | 0.236 |
| Urate-lowering therapy | 78 (23.0%) | 47 (13.8%) | 73 (21.1%) | 0.006 |
| Gout | 42 (12.4%) | 49 (14.4%) | 71 (20.5%) | 0.010 |
| Body mass index (kg/m^2^) | 23.4 ± 2.5 | 23.7 ± 2.5 | 23.5 ± 2.6 | 0.280 |
| Systolic blood pressure (mmHg) | 130.2 ± 17.6 | 132.6 ± 18.2 | 137.7 ± 19.3 | <0.001 |
| Diastolic blood pressure (mmHg) | 80.2 ± 14.2 | 80.0± 14.4 | 80.2 ± 15.2 | 0.982 |
| eGFR (mL/min/1.73 m^2^) | 32.7 ± 13.1 | 32.2 ± 15.0 | 26.9 ± 14.4 | <0.001 |
| Urinary albumin (mg/g Cr) | 1030.1 ± 1019.3 | 1270.9 ± 1196.0 | 1359.1 ± 1250.9 | 0.001 |
| Albumin (g/dL) | 4.2 ± 0.4 | 4.1 ± 0.4 | 4.0 ± 0.5 | 0.007 |
| Calcium (mg/dL) | 9.1 ± 0.4 | 9.1 ± 0.4 | 9.0 ± 0.4 | 0.231 |
| Phosphate (mg/dL) | 3.6 ± 0.8 | 3.6 ± 0.8 | 4.0 ± 0.9 | <0.001 |
| Total cholesterol (mg/dL) | 209.8 ± 39.9 | 209.8 ± 42.5 | 206.9 ± 39.1 | 0.556 |
| Hemoglobin (g/dL) | 12.0 ± 2.0 | 11.7 ± 2.1 | 11.3 ± 2.0 | <0.001 |
| CRP (mg/dL) | 0.6 ± 0.5 | 0.7 ± 0.6 | 0.8 ± 0.6 | 0.031 |
| Intact PTH (pg/mL) | 82.7 ± 63.8 | 99.1 ± 78.5 | 127.4 ± 86.6 | <0.001 |
| LVMI (g/m^2^) | 87.5 ± 18.3 | 101.4 ± 24.2 | 115.0 ± 22.7 | <0.001 |
| E (cm/s) | 63.8 ± 8.5 | 61.1 ± 7.7 | 60.0 ± 9.3 | <0.001 |
| e' (cm/s) | 8.6 ± 1.3 | 7.6 ± 1.2 | 6.9 ± 1.0 | <0.001 |
| E/e' | 7.6 ± 1.4 | 8.3 ± 1.6 | 8.8 ± 1.8 | <0.001 |
| Left atrial volume index (mL/m^2^) | 30.0 ± 6.4 | 35.7 ± 6.7 | 38.9 ± 4.5 | <0.001 |
| LVEF (%) | 61.6 ± 7.1 | 61.6 ± 6.9 | 61.7 ± 7.4 | 0.950 |
| LVH | 41 (12.1%) | 139 (40.9%) | 245 (70.8%) | <0.001 |
| LVDD | 91 (26.8%) | 239 (70.3%) | 327 (94.5%) | <0.001 |

Data are presented as mean ± standard deviation or (n, %). ^a^Coronary heart disease is defined as a history of coronary artery bypass surgery or percutaneous transluminal coronary angioplasty. ^b^Cerebrovascular disease is defined as a history of stroke or transient ischemic attack. ^c^P indicates statistical significance between the 3 groups. ACEI, angiotensin-converting enzyme inhibitor; ARB, angiotensin receptor blocker; CKD, chronic kidney disease; CRP, C-reactive protein; E, early mitral inflow velocity; e', peak early mitral annular velocity, eGFR, estimated glomerular filtration rate; PTH, parathyroid hormone; LVDD, left ventricular diastolic dysfunction; LVEF, left ventricular ejection fraction; LVH, left ventricular hypertrophy; LVMI, left ventricular mass index.

🡪Patients with a higher tertile of SUA have higher levels of left atrial volume index.

**Table ~~3~~4. Univariable and multivariable analyses for variables associated with LVH in study population (n = 1025).**

|  | **Univariable** | | **Multivariable** | |
| --- | --- | --- | --- | --- |
|  | Odds ratio (95% CI) | P | Odds ratio (95% CI) | P |
| Age (1 year) | 1.04 (1.03–1.05) | <0.001 | ~~1.03 (1.02–1.05)~~  1.03 (1.01–1.05) | <0.001 |
| Sex, male | 0.95 (0.74–1.21) | 0.658 | ~~0.99 (0.73–1.36)~~  0.99 (1.00–0.73) | ~~0.969~~  0.991 |
| Diabetes | 0.86 (0.67–1.10) | 0.231 | ~~0.94 (0.69-1.28)~~  0.91 (0.67–1.25) | ~~0.692~~  0.557 |
| Cardiovascular disease |  |  |  |  |
| Coronary heart disease^a^ | 1.55 (1.15–2.10) | 0.005 | ~~1.45 (0.99–2.11)~~  1.52 (1.04–2.25) | ~~0.055~~  0.033 |
| Cerebrovascular disease^b^ | 1.42 (0.96–2.10) | 0.083 |  |  |
| Peripheral vascular disease | 1.24 (0.78–1.95) | 0.366 |  |  |
| Medication |  |  |  |  |
| ACEI or ARB | 0.92 (0.69–1.24) | 0.595 |  |  |
| Calcium channel blocker | 1.15 (0.89–1.49) | 0.296 |  |  |
| Beta blocker | 1.08 (0.84–1.40) | 0.539 |  |  |
| Diuretics (thiazide) | 0.79 (0.59–1.04) | 0.091 |  |  |
| Diuretics (loop) | 1.15 (0.90–1.48) | 0.264 |  |  |
| Urate-lowering therapy | 1.26 (0.92–1.72) | 0.153 |  |  |
| Gout | 1.47 (1.05–2.05) | 0.026 | ~~1.06 (0.69–1.62)~~  1.08 (0.70–1.67) | ~~0.804~~  0.723 |
| Body mass index (1 kg/m^2^) | 0.97 (0.93–1.03) | 0.315 |  |  |
| Systolic blood pressure (10 mmHg) | 1.33 (1.23–1.43) | <0.001 | ~~1.25 (1.13–1.39)~~  1.24 (1.11–1.38) | <0.001 |
| Diastolic blood pressure (10 mmHg) | 1.11 (1.02–1.21) | 0.016 | ~~0.90 (0.79–1.02)~~  0.89 (0.78–1.01) | ~~0.104~~  0.068 |
| eGFR (1 ml/min/1.73 m^2^) | 0.97 (0.96–0.98) | <0.001 | ~~1.01 (0.99–1.02)~~  1.00 (0.99–1.02) | ~~0.560~~  0.663 |
| Urinary albumin (100 mg/g Cr) | 1.02 (1.01–1.03) | 0.002 | ~~1.01 (0.97–1.05)~~  1.01 (0.97–1.06) | ~~0.695~~  0.622 |
| Albumin (1 g/dL) | 0.71 (0.53–0.95) | 0.023 | ~~1.22 (0.40–3.67)~~  1.33 (0.43–4.14) | ~~0.727~~  0.622 |
| Uric acid (1 mg/dL) | 1.56 (1.47–1.65) | <0.001 | ~~1.51 (1.42–1.61)~~  1.40 (1.31–1.50) | <0.001 |
| Calcium (1 mg/dL) | 0.88 (0.64–1.21) | 0.427 |  |  |
| Phosphate (1 mg/dL) | 1.47 (1.27–1.71) | <0.001 | ~~0.94 (0.76–1.16)~~  0.93 (0.75–1.15) | 0.584  0.495 |
| Total cholesterol (1 mg/dL) | 1.00 (1.00–1.00) | 0.205 |  |  |
| Hemoglobin (1 g/dL) | 0.85 (0.80–0.91) | <0.001 | ~~1.00 (0.91–1.10)~~  1.00 (0.91–1.10) | ~~0.981~~  1.000 |
| CRP (1 mg/dL) | 1.44 (1.15–1.80) | 0.001 | ~~1.07 (0.82–1.39)~~  1.08 (0.83–1.42) | ~~0.625~~  0.568 |
| Intact PTH (10 pg/mL) | 1.09 (1.07–1.11) | <0.001 | 1.06 (1.03–1.09) | <0.001 |
| Left atrial volume index (1 mL/m^2^) | 1.16 (1.13–1.18) | <0.001 | 1.08 (1.50–1.11) | <0.001 |

^a^Coronary heart disease is defined as a history of coronary artery bypass surgery or percutaneous transluminal coronary angioplasty. ^b^Cerebrovascular disease is defined as a history of stroke or transient ischemic attack. ACEI, angiotensin-converting enzyme inhibitor; ARB, angiotensin receptor blocker; CI, confidence interval; CRP, C-reactive protein; eGFR, estimated glomerular filtration rate; PTH, parathyroid hormone; LVH, left ventricular hypertrophy.

🡪Univariable and multivariable analysis showed that left atrial volume index was significantly associated with LVH

**Table ~~4~~5. Univariable and multivariable analyses for variables associated with LVDD in study population (n = 1025).**

|  | **Univariable** | | **Multivariable** | |
| --- | --- | --- | --- | --- |
|  | Odds ratio (95% CI) | P | Odds ratio (95% CI) | P |
| Age (1 year) | 1.02 (1.00–1.03) | 0.012 | ~~1.01 (0.99–1.02)~~  0.99 (0.95–1.03) | ~~0.397~~  0.619 |
| Sex, male | 0.99 (0.77–1.28) | 0.949 | ~~0.97 (0.69–1.35)~~  1.48 (0.63–3.48) | ~~0.841~~  0.365 |
| Diabetes | 1.08 (0.84–1.39) | 0.566 | ~~1.26 (0.90–1.76)~~  1.62 (0.67–3.92) | ~~0.177~~  0.288 |
| Cardiovascular disease |  |  |  |  |
| Coronary heart disease^a^ | 1.13 (0.83–1.56) | 0.439 | 1.74 (0.57–5.25) | 0.328 |
| Cerebrovascular disease^b^ | 1.10 (0.73–1.67) | 0.645 |  |  |
| Peripheral vascular disease | 1.18 (0.72–1.92) | 0.509 |  |  |
| Medication |  |  |  |  |
| ACEI or ARB | 0.98 (0.73–1.33) | 0.918 |  |  |
| Calcium channel blocker | 1.10 (0.85–1.43) | 0.483 |  |  |
| Beta blocker | 0.97 (0.74–1.25) | 0.786 |  |  |
| Diuretics (thiazide) | 0.90 (0.68–1.20) | 0.472 |  |  |
| Diuretics (loop) | 1.00 (0.77–1.29) | 1.000 |  |  |
| Urate-lowering therapy | 0.88 (0.64–1.21) | 0.418 |  |  |
| Gout | 1.18 (0.83–1.69) | 0.357 |  |  |
| Body mass index (1 kg/m^2^) | 0.98 (0.93–1.04) | 0.498 |  |  |
| Systolic blood pressure (10 mmHg) | 1.22 (1.13–1.30) | <0.001 | ~~1.21 (1.08–1.35)~~  1.42 (1.05–1.92) | ~~0.001~~  0.023 |
| Diastolic blood pressure (10 mmHg) | 1.14 (1.05–1.25) | 0.003 | ~~0.95 (0.82–1.09)~~  0.69 (0.47–0.99) | ~~0.434~~  0.055 |
| eGFR (1 mL/min/1.73 m^2^) | 0.99 (0.98–1.00) | 0.010 | ~~1.01 (0.99–1.03)~~  1.01 (0.97–1.06) | ~~0.255~~  0.554 |
| Urinary albumin (100 mg/g Cr) | 1.01 (1.00–1.02) | 0.070 |  |  |
| Albumin (1 g/dL) | 0.83 (0.60–1.12) | 0.221 |  |  |
| Uric acid (1 mg/dL) | 1.98 (1.82–2.16) | <0.001 | ~~2.00 (1.83–2.19)~~  1.93 (1.53–2.43) | <0.001 |
| Calcium (1 mg/dL) | 0.90 (0.65–1.24) | 0.513 |  |  |
| Phosphate (1 mg/dL) | 1.26 (1.08–1.48) | 0.003 | ~~0.94 (0.75–1.19)~~  0.64 (0.35–1.17) | ~~0.944~~  0.144 |
| Total cholesterol (1 mg/dL) | 1.00 (1.00–1.00) | 0.662 |  |  |
| Hemoglobin (1 g/dL) | 0.94 (0.88–1.00) | 0.047 | ~~1.06 (0.96–1.18)~~  0.97 (0.74–1.27) | ~~0.263~~  0.798 |
| CRP (1 mg/dL) | 1.15 (0.92–1.45) | 0.230 |  |  |
| Intact PTH (10 pg/mL) | 1.05 (1.03–1.07) | <0.001 | ~~1.02 (0.99–1.05)~~  1.01 (0.94–1.09) | ~~0.273~~  0.711 |
| Left atrial volume index (1 mL/m^2^) | 3.74 (2.90–4.81) | <0.001 | 3.09 (2.45–3.90) | <0.001 |

^a^Coronary heart disease is defined as a history of coronary artery bypass surgery or percutaneous transluminal coronary angioplasty. ^b^Cerebrovascular disease is defined as a history of stroke or transient ischemic attack. ACEI, angiotensin-converting enzyme inhibitor; ARB, angiotensin receptor blocker; CI, confidence interval; CRP, C-reactive protein; eGFR, estimated glomerular filtration rate; PTH, parathyroid hormone; LVDD, left ventricular diastolic dysfunction.

🡪Univariable and multivariable analysis showed that left atrial volume index was significantly associated with LVDD

🡪We added correlation between SUA and left atrial volume index in Figure 1 as follows

There are significant correlation between SUA and left atrial volume index.

**Fig 1. Correlations between SUA levels and LVMI (A), e' (B), E/e' (C), and left atrial volume index (D) in patients with pre-dialysis CKD (n = 1025).** The SUA level correlated positively with the LVMI, E/e' ratio, and left atrial volume index and correlated negatively with the e'. CKD, chronic kidney disease; E, early mitral inflow velocity; e', peak early mitral annular velocity; LVMI, left ventricular mass index; SUA, serum uric acid.

**MINOR CONCERNS**

Material and Methods
There is a need to define, even if briefly, the stages of Chronic Kidney Disease (CKD).

Answer) As you suggested, we added definition of the stages of CKD as follows.

Depending on the eGFR value, each patient was classified into one of the 3 CKD groups: CKD stage 3 (n = 511), 30 ≤ eGFR < 60 mL/min/1.73 m^2^; CKD stage 4 (n = 356), 15 ≤ eGFR <30 mL/min/1.73 m^2^; CKD stage 5 (n = 158), eGFR < 15 mL/min/1.73 m^2^.

Line 105:
According to the American Society of Echocardiography Recommendations for the Evaluation of Left ventricular Diastolic Function Guidelines (Nagueh SF et al. J Am Soc Echocardiogr 2016;29:277-314) the correct is e´, instead of E´.

Answer) We corrected E´ to e´.

Lines 106-108:
“The E/E' ratio was calculated and used for the estimation of LV filling pressure. The severity of diastolic dysfunction was assessed using the E' values and E/E' ratios [13]. According to guideline of the American Society of Echocardiography [14]”.
It seems more appropriate to rewritten as follows:
“The E/e' ratio was calculated and used for the estimation of LV filling pressure. The severity of diastolic dysfunction was assessed using the e' values and E/e' ratio, [13] according to the American Society of Echocardiography Guidelines for the Evaluation of Left Ventricular diastolic Function. [14]”

Answer) We revised as you suggested

Lines 108-110:
”According to guideline of the American Society of Echocardiography [14], E', left atrium volume, E/A, deceleration time of E, and E/E' was used to categorize diastolic dysfunction into normal function, or grades 1, 2, or 3 diastolic dysfunction.”
It seems more suitable to rewritten as follows:
“...was used to categorize diastolic function into normal or grades 1, 2,or 3....”

Answer) We revised as you suggested

Lines 233-234:
“Notably, combination of SUA, SBP, and intact PTH) exhibited AUC of 0.836 (95% CI: 0.812–0.860), which was significant higher than that of SUA alone (0.836 vs. 0.803, P < 0.00”
There is no parenthesis after PTH....Which was significantly higher than...

Answer) We revised as you suggested

**Reviewer #2**

Dear Authors, this is a very nice work with more than 1000 patients. The association of uric acid with LVH or LVDD is unclear and for sure research has to be done. It is confused if LVH and diastolic dysfunction come only from the increased SBP and the renal failure, but the statistical analysis you provide gives strong support for the role of UA. I would like to ask why you didn't exclude CAD patients as well as it is established that many of these patients will also have diastolic dysfunction at baseline. Also, did you try to match your subgroups according to SBP which showed significant correlation with LVH and LVDD in multi-variable analysis? Finally, in tables 1 & 2 the p values refer to the overall comparison between the subgroups? The use of M-Mode for LV mass assessment seems a bit outdated but we can accept it as it in the ASE guidelines.
Language is proper
In PubMed, there are a lot of publications for the role of SUA in LVH and LVDD. Is the content of your manuscript so strong to add valuable data in the literature? It is important that you have included so many patients and even if there are a lot similar publications yours may add experience in this field and raise our awareness for research.
Thank you for your submission.

Comment 1) I would like to ask why you didn't exclude CAD patients as well as it is established that many of these patients will also have diastolic dysfunction at baseline.

Answer: We did not excluded CAD patients (n = 214) because loss of baseline data was severe if the CAD patients were excluded. Of the CAD patients (n = 214), 142 patients (66.4%) had the diastolic dysfunction at baseline. Thus, instead of excluding the CAD patients, we included the CAD patients in multivariable analysis for adjusting the effect of CAD on LVH and LVDD.

Comment 2) Also, did you try to match your subgroups according to SBP which showed significant correlation with LVH and LVDD in multi-variable analysis

Answer) We add a table (Baseline characteristics of the study population according to tertiles of systolic blood pressure values) as follows.

**Table 3. Baseline characteristics of the study population according to tertiles of systolic blood pressure values (n = 1025).**

|  | **Tertile 1**  **(<129 mmHg)** | **Tertile 2**  **(129-141 mmHg)** | **Tertile 3**  **(>141 mmHg)** | **P**^c^ |
| --- | --- | --- | --- | --- |
| Age (years) | 59.3 ± 10.7 | 60.9 ± 10.5 | 60.6 ± 10.2 | 0.109 |
| Sex, male [n (%)] | 180 (53.4%) | 170 (48.0%) | 172 (51.5%) | 0.355 |
| Diabetes [n (%)] | 182 (53.7%) | 171 (50.3%) | 169 (48.8%) | 0.430 |
| Cardiovascular disease [n (%)] |  |  |  |  |
| Coronary heart disease^a^ | 73 (21.7%) | 61 (17.2%) | 80 (24.0%) | 0.087 |
| Cerebrovascular disease^b^ | 34 (10.1%) | 33 (9.3%) | 45 (13.5%) | 0.182 |
| Peripheral vascular disease | 23 (6.8%) | 25 (6.9%) | 33 (9.9%) | 0.227 |
| Medication [n (%)] |  |  |  |  |
| ACEI or ARB | 257 (76.3%) | 271 (76.6%) | 250 (74.9%) | 0.857 |
| Calcium channel blocker | 202 (59.9%) | 222 (62.7%) | 223 (63.1%) | 0.283 |
| Beta blocker | 132 (39.2%) | 131 (38.9%) | 145 (41.3%) | 0.511 |
| Diuretics (thiazide) | 113 (33.5%) | 92 (26.0%) | 82 (24.6%) | 0.020 |
| Diuretics (loop) | 156 (46.3%) | 143 (40.4%) | 155 (46.4%) | 0.189 |
| Urate-lowering therapy | 61 (18.1%) | 73 (20.6%) | 64 (19.2%) | 0.701 |
| Gout | 50 (14.8%) | 54 (15.3%) | 58 (17.4%) | 0.628 |
| Body mass index (kg/m^2^) | 23.7 ± 2.5 | 23.5 ± 2.5 | 23.4 ± 2.3 | 0.293 |
| eGFR (mL/min/1.73 m^2^) | 35.6 ± 13.9 | 30.4 ± 13.6 | 25.7 ± 14.0 | <0.001 |
| Urinary albumin (mg/g Cr) | 1038.8 ± 1076.8 | 1321.2 ± 1246.8 | 1298.6 ± 1150.4 | 0.002 |
| Albumin (g/dL) | 4.2 ± 0.4 | 4.1 ± 0.5 | 4.1 ± 0.4 | 0.009 |
| Calcium (mg/dL) | 9.1 ± 0.4 | 9.0 ± 0.4 | 9.0 ± 0.4 | 0.306 |
| Phosphate (mg/dL) | 3.6 ± 0.7 | 3.8 ± 0.9 | 3.9 ± 0.9 | <0.001 |
| Total cholesterol (mg/dL) | 209.2 ± 38.8 | 207.8 ± 41.1 | 209.6 ± 41.5 | 0.832 |
| Hemoglobin (g/dL) | 12.4 ± 1.9 | 11.5 ± 2.2 | 11.2 ± 1.9 | <0.001 |
| CRP (mg/dL) | 0.6 ± 0.4 | 0.7 ± 0.6 | 0.8 ± 0.7 | 0.019 |
| Intact PTH (pg/mL) | 78.7 ± 58.3 | 103.8 ± 77.7 | 127.4 ± 90.6 | <0.001 |
| LVMI (g/m^2^) | 95.2 ± 23.7 | 98.8 ± 23.5 | 110.4 ± 24.0 | <0.001 |
| E (cm/s) | 61.7 ± 8.3 | 61.3 ± 8.8 | 61.8 ± 9.0 | 0.722 |
| e' (cm/s) | 7.9 ± 1.3 | 7.7 ± 1.3 | 7.4 ± 1.3 | <0.001 |
| E/e' | 7.9 ± 1.5 | 8.1 ± 1.6 | 8.6 ± 1.8 | <0.001 |
| Left atrial volume index (mL/m^2^) | 33.7 ± 7.0 | 34.9 ± 6.9 | 36.5 ± 6.7 | <0.001 |
| LVEF (%) | 62.4 ± 7.1 | 61.3 ± 7.1 | 61.2 ± 7.1 | 0.041 |
| LVH | 100 (29.7%) | 128 (36.2%) | 197 (59.0%) | <0.001 |
| LVDD | 186 (55.2%) | 229 (64.7%) | 242 (72.5%) | <0.001 |

Data are presented as mean ± standard deviation or (n, %). ^a^Coronary heart disease is defined as a history of coronary artery bypass surgery or percutaneous transluminal coronary angioplasty. ^b^Cerebrovascular disease is defined as a history of stroke or transient ischemic attack. ^c^P indicates statistical significance between the 3 groups. ACEI, angiotensin-converting enzyme inhibitor; ARB, angiotensin receptor blocker; CKD, chronic kidney disease; CRP, C-reactive protein; E, early mitral inflow velocity; e', peak early mitral annular velocity, eGFR, estimated glomerular filtration rate; PTH, parathyroid hormone; LVDD, left ventricular diastolic dysfunction; LVEF, left ventricular ejection fraction; LVH, left ventricular hypertrophy; LVMI, left ventricular mass index.

🡪The baseline characteristics of the study population according to systolic blood pressure are shown in table 3. Patients with a higher tertile of systolic blood pressure were more likely to have higher levels of phosphate (P < 0.001), intact PTH (P < 0.001), LVMI (P < 0.001), E/e' (P < 0.001), and left atrial volume index (P < 0.001) and lower levels of eGFR (P < 0.001), hemoglobin (P < 0.001), e' (P < 0.001), and LVEF (P = 0.041). They were also likely to have a higher prevalence of LVH (P < 0.001) and LVDD (P < 0.001).

Comment 3) Finally, in tables 1 & 2 the p values refer to the overall comparison between the subgroups?

Answer) p value refer to the ovrall comparison between the 3 groups. We added the phrase “P indicates statistical significance between the 3 groups.” in table 1 and 2.
