# Supplementary material for: Association between serum uric acid and left ventricular hypertrophy/left ventricular diastolic dysfunction in patients with chronic kidney disease
Source: PLoS One. 2021 May 6;16(5):e0251333. doi: 10.1371/journal.pone.0251333 (PMC8101764; doi:10.1371/journal.pone.0251333)
Supplement: Attachment — Submitted filename: Response to Reviewers.docx [file pone.0251333.s002.docx]

**PONE-D-21-04044R1
Association between serum uric acid and left ventricular hypertrophy/left ventricular diastolic dysfunction in patients with chronic kidney disease**

**Reviewer #1:** Dear Authors

Thank you for resubmitting your study addressing the recommendations/suggestions made in the former review.

I consider that all the issues asked by me were properly answered, except for the following:
Line 246: “... CI: 0.812–0.860), which was significant higher than that of SUA alone (0.836 vs. 0.803, P < 0.001).”
The correct is “....significantly higher than....”

Answer) As you commented, we corrected “significant” to “significantly”
